# Engineering Infrequent DNA Nicking Endonuclease by Fusion of a *Bam*HI Cleavage-Deficient Mutant and a DNA Nicking Domain

**DOI:** 10.3389/fmicb.2021.787073

**Published:** 2022-02-01

**Authors:** Shuang-yong Xu

**Affiliations:** New England Biolabs, Inc., Ipswich, MA, United States

**Keywords:** *Bam*HI catalytic mutant, phage Gamma HNH nicking domain, infrequent nicking enzyme, protein engineering, DNA nicking assay

## Abstract

Strand-specific DNA nicking endonucleases (NEases) typically nick 3–7 bp sites. Our goal is to engineer infrequent NEase with a >8 bp recognition sequence. A *Bam*HI catalytic-deficient mutant D94N/E113K was constructed, purified, and shown to bind and protect the GGATCC site from *Bam*HI restriction. The mutant was fused to a 76-amino acid (aa) DNA nicking domain of phage Gamma HNH (gHNH) NEase. The chimeric enzyme was purified, and it was shown to nick downstream of a composite site 5′ GGATCC-N(4-6)-AC↑CGR 3′ (R, A, or G) or to nick both sides of *Bam*HI site at the composite site 5′ CCG↓GT-N5-GGATCC-N5-AC↑CGG 3′ (the down arrow ↓ indicates the strand shown is nicked; the up arrow↑indicates the bottom strand is nicked). Due to the attenuated activity of the small nicking domain, the fusion nickase is active in the presence of Mn^2+^ or Ni^2+^, and it has low activity in Mg^2+^ buffer. This work provided a proof-of-concept experiment in which a chimeric NEase could be engineered utilizing the binding specificity of a Type II restriction endonucleases (REases) in fusion with a nicking domain to generate infrequent nickase, which bridges the gap between natural REases and homing endonucleases. The engineered chimeric NEase provided a framework for further optimization in molecular diagnostic applications.

## Introduction

In nature, DNA nicking endonucleases (NEases) are mainly involved in DNA restriction, rolling circle replication, F plasmid transfer, and DNA damage repair (Zheleznaya et al., 2009; Chan et al., 2011; Xu, 2015). The restriction endonuclease (REase)-derived nicking enzymes typically nick DNA target sites in 3–7 bp, and homing NEases recognition sequences are usually more than 16 bp (Chan et al., 2011). Therefore, there is a significant gap of NEases that nick DNA sites in the range of 8–15 bp. To expand the DNA target sequence range, we propose to utilize the DNA binding specificities of Type II REases (3–8 bp) that are cleavage-deficient with mutations in the catalytic site and fusion of the mutant enzymes to a DNA nicking domain [e.g., phage Gamma HNH (gHNH) nicking endonuclease, gHNH, 3–5 bp recognition (Xu and Gupta, 2013)]. When a REase binding specificity for 7–8 bp target is fused to a gHNH nicking domain of 5 bp (e.g., ACCGR, complementary strand YCGGT, R = A or G, Y = C or T), it is possible to generate an infrequent nicking enzyme with 12–13 bp composite sites. It is shown that the minimal DNA nicking domain of the gHNH endonuclease contains only 76 amino acid (aa) residues and required Mn^2+^ for stimulated nicking activities (Xu and Gupta, 2013).

For isothermal DNA amplification, thermostable nicking enzymes are needed to be compatible with Bst DNA polymerase active at 50–65°C. Thermostable DNA NEase has been found in thermophilic phage genome isolated from deep sea vent (Xu and Gupta, 2013) and a large number of metagenome sequences from deep sea vent and hot springs can be rich sources for discovery of more thermostable NEases (Zhang et al., 2017; Castelan-Sanchez et al., 2019). Thermostable NEases, such as Nt.BstNBI and Nb.*Bsr*DI, are part of natural occurring restriction systems. When they are combined with the corresponding small subunits, they become double-stranded (ds) DNA cleaving enzymes (Higgins et al., 2001; Xu et al., 2007).

Sequence-specific DNA NEases have been used in DNA optical mapping by nicking, nick translation of the nicked sites with fluorescently labeled dNTP, and religation of the nicked strand (Zhang et al., 2010; Jeffet et al., 2021). Large chromosome rearrangement (insertion, deletion, or translocation) of cancer cells could be “visualized” under high-resolution fluorescent microscope when the abnormal DNA nicking pattern images are compared with the WT (English et al., 2015; Jeffet et al., 2021) (Bionano Genomics, Irys system). NEases are also used in isothermal DNA amplifications, such as strand-displacement DNA amplification (SDA) (Walker et al., 1994), EXPAR (Qian et al., 2012), nicking enzyme assisted amplification (NEAA) (Van Ness et al., 2003; Qian et al., 2019), Nt.CviPII-assisted random whole genome amplification (Chan et al., 2004), and in NicE-seq to study open chromatin profiling (Ponnaluri et al., 2017). NEase-mediated amplification is utilized in FDA-approved clinical diagnostic testing that enabled the first CLIA-waived molecular diagnostic test for rapid identification of influenza A and B. (Nie et al., 2014). Most recently, nicking enzyme-assisted reaction (NEAR) technology has been applied to the detection of COVID-19 viral RNA by reverse transcription, DNA nicking, and isothermal DNA amplification (James and Alawneh, 2020). NEAR (ID NOW™ COVID-19 (Abbott/Alere) was able to detect SARS-CoV2 genomic fragment in a short time (10 min).

In this work, we constructed and purified a catalytic-deficient *Bam*HI mutant D94N/E113K. It was shown previously that D94N or E113K single mutant displayed poor cleavage activity but maintained specific binding to *Bam*HI site in a DNA mobility shift assay (Xu and Schildkraut, 1991). Here, the purified *Bam*HI mutant D94N/E113K was shown to bind and protect against *Bam*HI digestion of a plasmid with a single *Bam*HI site. We then fused the *Bam*HI mutant to a small gHNH nicking domain and purified the fusion enzyme. The fusion nicked the composite site GGATCC-N(4-6)-AC↑CGR in NEB buffer 2 (50 mM NaCl) and buffer 3 (100 mM NaCl) supplement with Mn^2+^ or Ni^2+^. The fusion NEase also nicks the composite site CCG↓GT-N5-GGATCC-N5 AC↑CGG both upstream (5′) and downstream (3′), potentially generating a long overhang (20 bases) after melting of the duplex. This work demonstrates a proof of concept for fusion of a catalytic-deficient REase and phage gHNH nicking domain and opened the door for further optimization of infrequent NEases in future research and diagnostic applications.

## Materials and Methods

### Enzymes, Plasmid DNA, and DNA Sequencing

Restriction endonucleases, Nb.*Bsr*DI, Proteinase K, pBR322, and pUC19 were provided by New England Biolabs, Inc. (NEB). Synthetic gene blocks were purchased from Integrated DNA Technologies (IDT). Big-Dye Sanger sequencing kit was purchased from Thermo-Fisher/ABI. Restriction buffer compositions (1 ×): Buffer 2: 50 mM NaCl, 10 mM Tris–HCl, 10 mM MgCl_2_, and pH 7.9 at 25°C. Buffer 3: 100 mM NaCl, 50 mM Tris–HCl, 10 mM MgCl_2_, and pH 7.9 at 25°C. CutSmart buffer (buffer 4 plus BSA): 50 mM Potassium Acetate, 20 mM Tris-acetate, 10 mM Magnesium Acetate, 100 μg/ml BSA, and pH 7.9 at 25°C.

In some nicking reactions, Mn^2+^ or Ni^2+^ divalent cations were supplemented in NEB buffer 2 or 3 as indicated. DNA blunting kit and blunt/TA quick ligation kit were provided by NEB. *E*scherichia *coli* competent cells T7 Express (C2566) and NEB 10β were also from NEB.

Nicking reactions were usually carried out at 37°C for 30–60 min or as indicated in specific buffers. One forward and one reverse primer were used to sequence (map) the *Bam*HI site and nicking site(s) in pUC19 and the adjacent sequences. The primers were purchased from IDT and have the following sequences:

pUC-F61, 5′ CAGCTTGTCTGTAAGCGGATGCCG 3′.

pUC-R600, 5′ GGGCAGTGAGCGCAACGCAAT 3′.

The DNA strands of the pUC19 sequence near *Bam*HI site is artificially defined as top and bottom strand as following:

top strand: 5′-…AGAGGATCCCCGGGTACCGAGC…-3′.

bottom strand: 3′-…TCTCCTAGGGGCCCATGGCTCG… -5′.

### Gene Assembly/Cloning

Synthetic gene blocks were assembled into pTYB1 vector (*Nde*I and *Xho*I digested) using Gibson assembly kit (NEB) and the DNA inserts were selected by plasmid DNA transformation and the correct insert was verified by DNA sequencing. Plasmids were prepared by plasmid mini-preparation kits (Qiagen, Sigma, or NEB). DNASTAR Lasergene software was used for sequence editing and visualization of nicking sites (doublet peaks) in DNA run-off sequencing.

The aa sequences of D94N/E113K and phage gHNH nicking domain are shown below. No linker aa residues were inserted between *Bam*HI* and the gHNH nicking domain due to the flexible nature of the *Bam*HI C-terminal region (Newman et al., 1994).

>*Bam*HI D94N/E113K (GGATCC) 213 aa (N and K residues shown in red are the aa substitutions, predicted molecular mass = 24.57 kDa)

MEVEKEFITD EAKELLSKDK LIQQAYNEVK TSICSPI WPA TSKTFTINNT

EKNCNGVVPI KELCYTLLED TYNWYREKPL DILKLEK KKG GPINVYKEFI

ENSELKRVGM EFKTGNISSA HRSMNKLLLG LKHGE IDLAI ILMPIKQLAY

YLTDRVTNFE ELEPYFELTE GQPFIFIGFN AEAYNSNVPL IPKGSDGMSK

RSIKKWKDKV ENK

>*Bacillus* phage gHNH small nicking domain (76 aa)

-fyrskawer lreqalmrdk glclhcknnr kikva dmvdh iipikvdpsl

klklenlqsl cnpchnrkta edkkkyg

>WT *Bacillus* phage Gamma HNH endonuclease 127 aa (GenBank accession number YP_338236)

(the minimal nicking domain sequence is shown in underlined residues).

mkkplrpcce fhcynltrer

yceehrykek etqqdknryy drfkrdkest afyrskawer lreqalmrdk glclhcknnr kikvadmvdh iipikvdpsl klklenlqsl cnpchnrkta edkkkyg.

### Protein Expression and Purification

The IMPACT protein expression system, including pTYB1 vector and chitin beads, was provided by NEB. Target protein was cleaved from the intein-CBD fusion by DTT cleavage overnight and eluted from a chitin column (in some cases, a second DTT overnight cleavage step was carried out, and target protein was eluted to increase recovery yield). Enzymes were further purified by chromatography through a heparin column (5 ml HiTrap Heparin HP, GE Healthcare). Proteins were concentrated by low-speed centrifugation in protein concentrators (10 kDa cut-off) and resuspended in enzyme storage buffer (200 mM NaCl, 10 mM Tris–HCl, pH 7.5, 1 mM DTT, and 50% glycerol) and kept in a –20°C freezer. T7 Express cells carrying *Bam*HI mutant gene or *Bam*HI*-gHNH fusion gene in a plasmid was cultured at 37°C to late log phase and enzyme production was initiated by addition of 0.5 mM IPTG final concentration and cells were induced at 18°C overnight in a temperature-controlled shaker. Cells were lysed by sonication in a chitin column buffer (20 mM Tris–HCl, pH 8.5, and 0.5 M NaCl) at 4°C. To remove nucleic acids from enzymes, *Bam*HI*-gHNH fusion protein eluted from the chitin column was diluted in a buffer (0.3 M NaCl, 20 mM Tris–HCl, pH 7.9, 0.1 mM ETDA, and 1 mM DTT) and passed through HiTrap DEAE column (Fast flow, 5 ml). Enzymes were concentrated by low-speed centrifugation in protein concentrators and resuspended in enzyme storage buffer.

## Results

### Construction of *Bam*HI Mutant D94N/E113K

**FIGURE 1:**
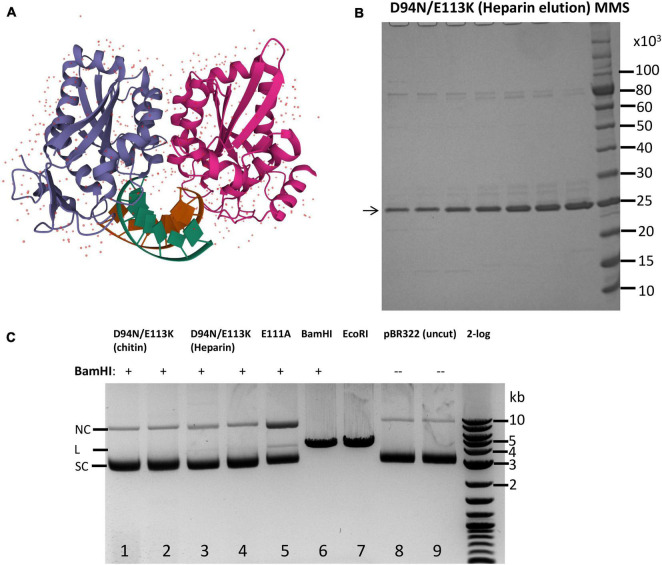
Purification of catalytic-deficient *Bam*HI mutant D94N/E113K and protection of *Bam*HI site by D94N/E113K against *Bam*HI restriction. **(A)** Crystal structure of a *Bam*HI dimer bound to the cognate site (tatGGATCCata) (PBD ID 1BHM). Two subunits of the *Bam*HI dimer are shown in purple and magenta colors, respectively. The DNA double helix is shown in green and brown colors. The small dots indicate water molecules in the dimer structure. **(B)** SDS-PAGE analysis of the purified *Bam*HI variant D94N/E113K (∼24.57 kDa). Eluted fractions from a heparin column (HiTrap Heparin 5 ml) were shown. MMS, protein molecular mass standard in Daltons (Da) (NEB). **(C)** *Bam*HI restriction digestion in the presence of D94N/E113K. Lanes 1–4, pBR322 (1 μg, ∼7.1 nM) with a single *Bam*HI site was first incubated with D94N/E113K (0.5 and 1 μg, ∼0.4 and 0.8 μM) at 25°C for 15 min, and 5 U of *Bam*HI was then added to the bound complex and incubated at 37°C for 30 min. After restriction digestion, Proteinase K (1.6 U) was added and incubated at 37°C for 15 min to remove *Bam*HI and the mutant protein. Lane 5 is the same as in lanes 1–4 except a different *Bam*HI catalytic mutant (E111A, 1 μg, ∼0.8 μM) was used. In lanes 6 and 7, pBR322 was linearized by *Bam*HI (10 U) and *Eco*RI (10 U), respectively, in the absence of D94N/E113K. Lanes 8, TE (10 mM Tris–HCl, 0.1 mM EDTA) mock digestion; lane 9, uncut pBR322. 2-log, DNA size ladder (100 bp to 10 kb, NEB). SC, supercoiled DNA; L, linear DNA; NC, nicked circular DNA. The size of linear pBR322 is ∼4.4 kb.

The structure of *Bam*HI endonuclease dimer in complex with cognate DNA had been solved previously (Figure 1A). The enzyme makes multiple direct hydrogen bonds and water-mediated hydrogen bonds with the G/C and A/T base pairs in the major groove in the recognition sequence GGATCC to form specific binding complex (Newman et al., 1994). In the cocrystal structure, the active site is formed by PD_94_-E_111_xE_113_ residues to bind divalent cations and active a water molecule for nucleophilic attack of the DNA backbone. A typical REase fold consisting of central four-stranded β-sheets flanked by two α-helices on both sides was present in *Bam*HI. The C-terminal *Bam*HI forms a flexible “arm” extension (Newman et al., 1995). The mechanism of REase and DNA interactions include five steps: (1) non-specific binding to dsDNA, (2) one-dimensional diffusion and two-dimensional hopping on DNA to locate a target site, (3) specific binding (hydrogen bonding to GGATCC sequence and DNA backbone), (4) enzyme activation in cleavage, and (5) product release (Aggarwal, 1995).

Prior to the crystal structure, random mutagenesis of the *bamHIR* gene identified three catalytic residues: E77, D94, and E113 because aa substitutions at these positions generated mutants (E77K, D94N, and E113K) with diminished catalytic activity (Xu and Schildkraut, 1991). Later, E111 was also identified as a catalytic residue by a genetic selection because E111A could bind to the *Bam*HI site and served as a “repressor” (Dorner and Schildkraut, 1994). However, E77K and E111A still showed a low residual cleavage activity at high enzyme concentration in overdigestion. To construct a *Bam*HI mutant with absolutely zero cleavage activity, we combined the two mutations to generate a double mutant D94N/E113K. The mutant enzyme was purified by chromatography through chitin and Heparin columns (Figure 1B). The partially purified protein was used to protect a single *Bam*HI site in pBR322 against *Bam*HI digestion. Figure 1C shows that the purified D94N/E113K protein protected pBR322 against *Bam*HI restriction (lanes 1–4). Purified E111A binding also protects the *Bam*HI site against *Bam*HI digestion although a small fraction of DNA was linearized, and some nicked circular DNA was also generated (lane 5). *Bam*HI alone digested pBR322 to linear DNA (lane 6). The mutant enzyme also blocked *Sma*I digestion of pUC19 because the *Bam*HI site overlaps with a *Sma*I site in GGATCCCGGG sequence (data not shown). No *Bam*HI cleavage activity was detected using 4 μg of the double mutant protein to digest pBR322 (data not shown) (4 μg of the WT *Bam*HI should have ∼2,000 U at ∼5 × 10^5^ U/mg specific activity) (Jack et al., 1991). We concluded from this preliminary experiment that *Bam*HI double mutant D94N/E113K did not show any cleavage activity, but it could bind to *Bam*HI site and protect the site against *Bam*HI restriction. The mutant protein can also protect *Sma*I site against *Sma*I digestion when *Bam*HI and *Sma*I sites overlap.

### Construction of *Bam*HI Catalytic Mutant and Gamma HNH Fusion

**FIGURE 2:**
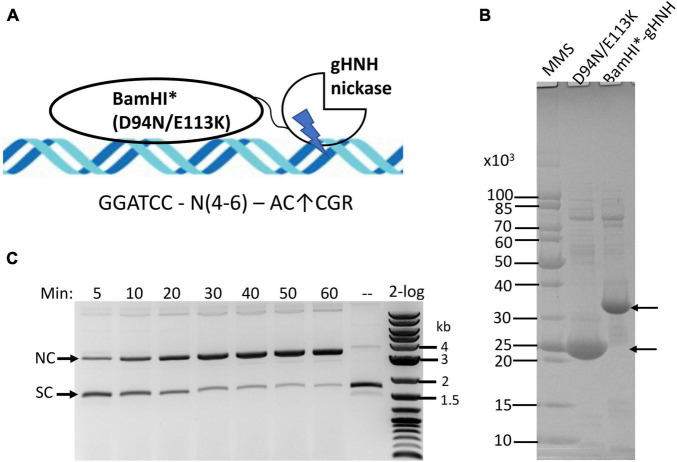
Purification of *Bam*HI catalytic mutant (*Bam*HI*)-gHNH fusion enzyme and nicking activity assay in the presence of divalent cations. **(A)** Schematic diagram of *Bam*HI catalytic mutant (*Bam*HI*) in fusion with phage gHNH nicking domain (the size of DNA and protein domains are not shown in scale). The diagram is simplified to show the monomeric fusion only. **(B)** SDS-PAGE analysis of partially purified *Bam*HI*-gHNH fusion protein. Lane 3, chitin column purified fusion protein (predicted molecular mass in ∼33.53 kDa). Following DTT cleavage overnight, eluants 1 and 2 from the chitin column were concentrated by low-speed centrifugation in centrifugal filter unit (Millipore, 10 kDa cut-off) and resuspended in an enzyme storage buffer. Approximately 20 and 5 μg of the *Bam*HI mutant and fusion proteins were loaded in the gel. MMS, protein molecular mass standard in Daltons (Da) (NEB). **(C)** A time course (5–60 min) of pUC19 nicking digestion (partial) by the fusion enzyme in NEB buffer 2 supplemented with MnCl_2_. Approximately 80% of supercoiled DNA was converted to nicked circular form after 1 h nicking reaction. A weak linear band also appeared. SC and NC, supercoiled and nicked circular DNA, respectively. 2-log, DNA size ladder (100 bp to 10 kb, NEB).

Our goal is to construct a chimeric NEase with infrequent nicking sites. The minimal phage gHNH nicking domain consists of 76-aa residues and has attenuated activity (the WT enzyme has 127-aa residues). The truncated nicking domain is more active in the presence of Mn^2+^ and it has poor activity in Mg^2+^ buffers (Xu and Gupta, 2013). A schematic diagram of the fusion construct is shown in Figure 2A. A synthetic gene block encoding *Bam*HI*-gHNH fusion (*Bam*HI* denotes D94N/E113K double mutant) was cloned into pTYB1 and the fusion enzyme was partially purified by chitin column chromatography and analyzed by SDS-PAGE (Figure 2B). The fusion enzyme yield is rather low, producing approximately 1 mg/L of IPTG-induced cells, which might be caused by the toxicity of the fusion enzyme as compared with the yield of ∼4 mg per L of IPTG-induced cells for the *Bam*HI double mutant (D94N/E113K) alone. The purified enzyme is active in nicking of pUC19 in a time course (5–60 min digestion) in NEB buffer 2 supplemented with 1 mM MnCl_2_ (Figure 2C). It is estimated that more than 80% of supercoiled DNA was converted to nicked circular form after 1 h digestion (under limited digestion condition). The fusion enzyme has relatively low nicking activity in standard restriction buffers with 10 mM MgCl_2_ (Supplementary Figure 1A). It is also active in Ni^2+^ buffer and the substrate DNA was partially nicked after 1 h digestion (Supplementary Figure 1B). In an enzyme titration experiment vs. fixed amount of pUC19 DNA, it was estimated that 1 μg of the fusion enzyme was required to completely nick 1 μg of the substrate into nicked circular DNA (Supplementary Figure 2). Therefore, the specific activity was estimated at ∼1–2 × 10^3^ U/mg protein (1 U is defined as the amount of enzyme required to completely nick 1 μg pUC19 into nicked circular form at 37°C for 1 h in NEB buffer 3 supplemented with 1 mM MnCl_2_). The full-length phage gHNH endonuclease is fully active in Mg^2+^ buffer and nicks the sites AC↑CGR (or AC↑CG in over-digestion) (Xu and Gupta, 2013). The specific nicking activity of gHNH endonuclease on pUC19 was estimated at 4–8 × 10^3^ U/mg in NEB buffer 3 (Supplementary Figure 2, bottom panel). The specific nicking activity of the short 76-aa nicking domain could not be determined due to its attenuated activity (estimated at less than 500 U/mg, Supplementary Figure 2, top panel). We conclude from this experiment that the activity of *Bam*HI*-gHNH fusion nickase was low in Mg^2+^ buffer, but its activity was stimulated in Mn^2+^ and Ni^2+^ buffers. Short incubation time (30–60 min) and medium to high salt are required for the chimeric enzyme to minimize non-specific (star) nicking activity (see below).

### Mapping of Nicking Sites in pUC19

We used DNA run-off sequencing to map the nicking site near the *Bam*HI binding sequence. It is based on the appearance of doublets in sequencing base calling. When the template strand is broken (nicked or cleaved), the sequencing reads with doublet A/C, A/G, A/T, or extra high A peak are generated by the template-independent terminal nucleotide transferase activity of the Taq DNA polymerase [or extra T peak in doublet (T/C, T/G, T/A) in reverse strand sequencing] (Too et al., 2010). One example of BspQI cleavage site mapping is shown in Supplementary Figure 3. BspQI recognition sequence is 5′ GCTCTTC 3′; but it cuts downstream at N1 (top strand)/N4 (bottom strand). When the bottom strand template was cleaved four nucleotides downstream of the site, Taq DNA polymerase “sensed” the broken template and added an extra “A” peak in the sequencing read (see a schematic diagram in panel A, and the actual sequencing read in panel B). If the cleavage/nicking reaction was partial (a mixture of uncut and cut templates), the run-off position would be indicated as a doublet (A/T). The top-strand cleavage position is marked by an extra “T” peak and sudden drop-off of sequencing peaks at the cleavage site. The bottom panel in Supplementary Figure 3C shows the control pBR322 (uncut) sequencing reactions in which the base calls are normally continuous and no apparent doublets were detected. Supplementary Figure 4 shows the run-off sequencing of *Bam*HI partially digested pUC19 and the appearance of T/G doublet in the cleaved *Bam*HI site 5′-G↓GATCC-3′.

**FIGURE 3:**
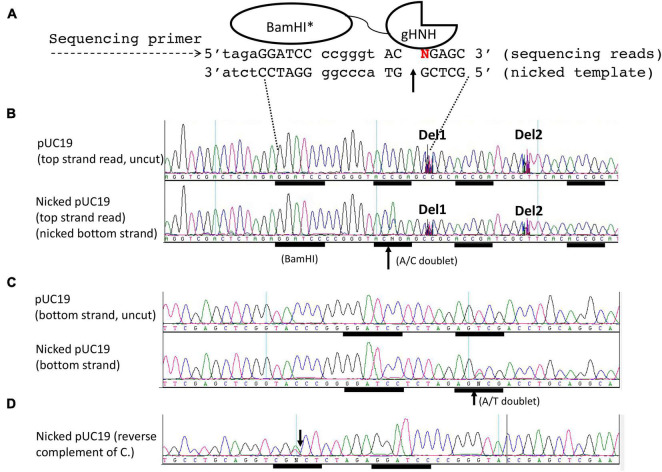
DNA run-off sequencing to map the nicking site(s) and nicking distance from a *Bam*HI site in pUC19. The plasmid substrate was digested by the fusion nickase at 37°C for 30 min in NEB buffer 2 supplemented with 1 mM MnCl_2_. **(A)** The composite nicking site GGATCC-N6-AC↑CGA is shown in a schematic diagram. **(B)** Top-strand sequencing read from the nicked bottom strand. The up arrow indicates the bottom strand of AC↑CGA was nicked and the appearance of an extra adenine (A) peak in the doublet as “A/C.” The addition of an extra peak “A” was catalyzed by the Taq DNA polymerase (template-independent terminal nucleotide transferase) when the template DNA is broken (nicked). Del1 and Del2 indicate some intervening sequences were manually deleted to show the downstream sites (ACCGA and ACCGC). **(C)** Bottom-strand sequencing read form nicked top-strand DNA. A star nicking site 5′ GGATCC N5 AGTCG 3′ was detected by the presence of an “A/T” doublet. The same sequence was shown as a reverse-complement in **(D)**. The down arrow indicates the nicked position.

We used the same run-off sequencing to determine the nicking site of the chimeric NEase as described above. The schematic diagram of the sequencing reaction is shown on the top of Figure 3A, in which the bottom strand serves as the template (nicked strand) and the top strand is the sequencing read. A doublet A/C is read as “N” by the sequence editing software, indicating bottom-strand nicking at GGATCC-N6-AC↑CGA (Figure 3B). In the uncut control DNA, the sequence reads as continuous bases and no doublet was detected (Figure 3B, top). It is noted that no cleavage or nicking occurred within the *Bam*HI site as one expects. In the bottom-strand sequencing, a nick (T/A doublet) is detected near the *Bam*HI site (GGATCC N5 AG↑TCG) (Figure 3C). The same sequence is shown as a reverse complement in Figure 3D (CGN↓CT N5 GGATCC). The nicking site AGTCG can be considered as a star nicking site of gHNH in Mn^2+^ buffer, which is somewhat analogous to ACCGG with the combined sequence of AS↑YSG (S = C or G, Y = C or T). Nicking at this star site was diminished in NEB buffer 3 supplemented with 1 mM MnCl_2_ or when this site is mutated to CTATA sequence (data not shown). DNA sequences ACCGA or ACCGC further downstream of the *Bam*HI site were not nicked. Some intervening sequences were manually deleted (as marked by Del1 and Del2) to show the downstream nicking sites. This mapping experiment showed that the fusion nicking enzyme recognizes GGATCC as an anchor site and nicks DNA 5–6 bp downstream (3′) or upstream (5′) in the cognate site ACCGR or a nearby site AGTCG. The stand-alone nicking sites (ACCGA, ACCGC, or other star sites) farther away from the *Bam*HI site are poor substrates for the fusion enzyme.

To analyze the nicking activity dependence on a *Bam*HI site, we deleted the *Bam*HI site in pUC19 by treatment of *Bam*HI-linearized plasmid with a blunting kit and religated the DNA and transferred the DNA into NEB 10β by transformation. Individual plasmids were prepared and confirmed the absence of *Bam*HI site by *Bam*HI digestion (data not shown). The pUC19-Δ*Bam*HI was used as a substrate for *Bam*HI*-gHNH nicking digestion. Only a small fraction of the DNA was nicked in 30 min digestion, and a majority of the DNA remained intact as supercoiled form (Supplementary Figure 5, lanes 1–4), suggesting weak non-specific nicking of star sites. In control digestions, pUC19-Δ*Bam*HI was resistant to *Bam*HI restriction, but it was linearized by *Eco*RI, and nicked completely by nicking enzyme Nb.*Bsr*DI. Nicked circular form of pUC19-Δ*Bam*HI was accumulated after long digestion (4 h to overnight) (data not shown), indicating possible non-specific nicking activity directed by the nicking domain or by some contaminating *E. coli* nucleases. In a control digestion, the fusion nickase completely nicked pUC19 in Mn^2+^ buffer although a small amount of linear DNA was also generated. We concluded from this experiment that efficient nicking of a plasmid required the presence of *Bam*HI site and gHNH nicking site. Some non-specific nicking may occur by the gHNH nicking domain during prolonged digestion.

### Mapping of Nicking Sites 4–5 bp Downstream of *Bam*HI Site

**FIGURE 4:**
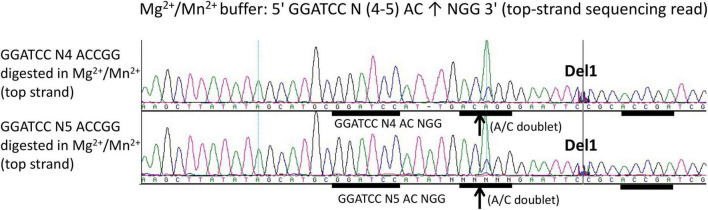
DNA run-off sequencing to map the nicking site and nicking distance from *Bam*HI in the engineered composite site GGATCC-N(4-5)-AC↑CGG in pUC19 (1xACCGG). Top-strand sequencing read using nicked bottom-strand template. DNA nicking reactions were carried out in NEB buffer 2 supplemented with Mn^2+^ (1 mM) at 37°C for 30 min. The up arrows indicate the nicked position where a strong A/C doublet was present. Del1 indicates the intervening sequence had been manually deleted to show the downstream site ACCGA (where no nicking had occurred). The composite sites GGATCC N4 ACCGG and GGATCC N5 ACCGG were inserted into pUC19 multiple cloning sites and flanked by *Hin*dIII and *Eco*RI sites. In addition, a star site (AGTCG) upstream of the *Bam*HI site was changed to the sequence TATAG to eliminate the star nicking activity.

To further map composite nicking sites, we constructed two pUC19 derivatives with GGATCC N(4–5) ACCGG and replace the star site AGTCG (complement CGACT) with CTATA (complement TATAG) upstream. The plasmids with one composite nicking site GGATCC N4 ACCGG or GGATCC N5 ACCGG were digested by the fusion enzyme and the nicked DNA was subjected to run-off sequencing. Figure 4 shows that only the downstream site AC↑CGG was nicked with a spacer N4 (top panel) or N5 (bottom panel) as a strong doublet (A/C) was detected. No apparent nicking was detected on the site ACCGA farther downstream or at the sequence TATAG (CTATA) upstream (data not shown). We concluded from this experiment that efficient DNA nicking required both a *Bam*HI site and a gHNH nicking site. No nicking was detected upstream at TATAG (complement CTATA) after replacing the star nicking site. However, we have not analyzed all possible star sites for the gHNH nicking domain in Mn^2+^ buffer.

### Mapping of Nicking Sites Near GGATCC 5 Nucleotides Upstream and Downstream

**FIGURE 5:**
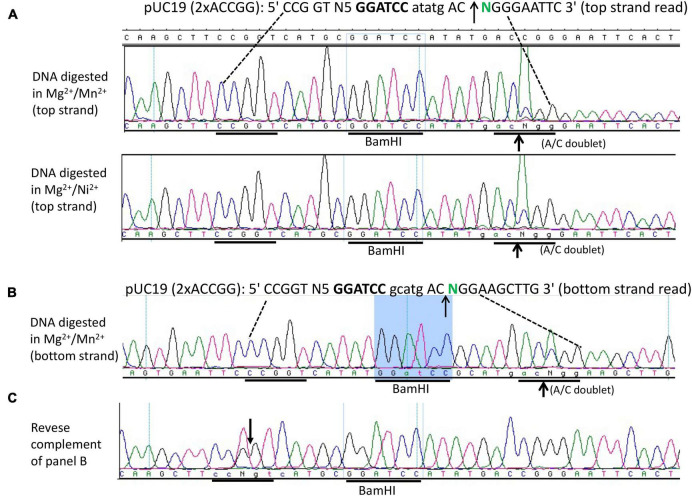
DNA run-off sequencing of the nicking sites and nicking distance from the *Bam*HI site. **(A)** Top-strand sequencing read from nicked bottom strand. Nicking reactions were carried out in NEB buffer 2 supplemented with MnCl_2_ (1 mM) or NiCl_2_ (1 mM) using pUC19 (2xACCGG) containing the composite site 5′ CCGGT-N5-GGATCC-N5 ACCGG 3′. The duplex oligos containing the composite site was inserted into pUC19 and flanked by *Hin*dIII (AAGCTT) and *Eco*RI (GAATTC) sites. **(B)** Bottom-strand sequencing read (nicked top-strand template) near the *Bam*HI site. The composite site contains a nick as indicated by a strong A/C doublet. If the bottom strand is shown as a reverse-complement, a T/G doublet would be present **(C)** in the nicking site.

We next constructed a symmetric composite site CCGGT N5 GGATCC N5 ACCGG in a pUC19 derivative and examined the fusion enzyme nicking activity in NEB buffer 2 supplemented with Mn^2+^. Figure 5 shows that the enzyme can nick the symmetric substrate both upstream and downstream (CCG↓GT N5 GGATCC N5 AC↑CGG), generating a long 5′ overhang of 20 nucleotides (nt) if the duplex is melted at high temperature. Such a long overhang might be useful for DNA cloning and DNA fragment end labeling. Figure 5A (sequencing panels 1 and 2) shows the nicking activity generated in the Mg^2+^/Mn^2+^ and Mg^2+^/Ni^2+^ buffers (i.e., top-strand sequencing read from nicked bottom-strand template). The bottom strand also contains a nick near the *Bam*HI site (Figure 5B) as evidenced by the presence of an A/C doublet, indicating both strands were nicked by the fusion nickase. The same sequence is shown in Figure 5C as a reverse complement (T/G doublet at the nicked position). Due to limited digestions to minimize star activity, the plasmid substrate was only partially nicked and did not reach complete nicking.

We next tested the fusion enzyme nicking activity in high salt buffers (100 mM to 200 mM NaCl + KCl) supplemented with 1 mM MnCl_2_ under limited digestion. The enzyme is partially active in the high salt buffers as shown in Supplementary Figure 6 although small inhibition was observed in 200 mM salt. The nicking site in pUC19 was mapped to the sequence 5′ GGATCC N6 AC↑CGA 3′.

## Discussion

In this work, we constructed an infrequent NEase by fusion of cleavage-deficient *Bam*HI mutant D94N/E113K and a phage gHNH nicking domain (∼76 aa). The fusion enzyme nicks near the *Bam*HI site N4-6 when a nicking site ACCGR/YCGGT is present either downstream or upstream or on both sides. The activity on the gHNH nicking site ACCGR or its variants alone is rather low, and no doublets were detected in run-off sequencing of ACCGR sites further downstream or upstream under limited digestion, which is likely due to the attenuated activity of the small nicking domain that requires a strong DNA binding partner to anchor onto DNA. The nicking site recognition could be relaxed to nick star sites (ASYSR) in Mn^2+^ buffer, which is consistent with the star activity of the full-length gHNH endonuclease in Mn^2+^ buffer (1–2 base off from ACCGR sites) (Xu and Gupta, 2013). To minimize the star activity of the fusion enzyme, high salt buffer (100–200 mM NaCl or KCl) may be required in digestion in Mg^2+^/Mn^2+^ buffer or Ni^2+^ buffer. The fusion enzyme is active in nicking reactions with 50–150 mM NaCl (200 mM NaCl slightly inhibits activity). The fusion NEases is active in Mn^2+^ or Ni^2+^ buffer, and it has low nicking activity in the presence of 10 mM Mg^2+^. To study the nicking site profile of gHNH minimal nicking domain in Mn^2+^ and Ni^2+^ buffers, it would be necessary to construct NGS libraries from nicked DNA and sequencing the inserts and map the sites back to the undigested DNA by examination of thousands of sites. The divalent cation Mg^2+^ is probably not required for specific binding to the *Bam*HI site for the D94N/E113K mutant as the single mutant D94N or E113K can bind to DNA in the absence of divalent cations (Xu and Schildkraut, 1991). For most of REases, they do not require divalent cations for binding, but for *Eco*RV-specific binding to the cognate site (GATATC), a divalent cation is required (Vipond et al., 1995). For example, Ca^2+^ supports binding but not cleavage for *Eco*RV on cognate DNA substrate.

A number of cleavage-deficient REases have been isolated before as reported in the literature (e.g., *Eco*O109I, *Eco*RI, *Eco*RV, *Bso*BI, BspQI/*Sap*I, *Hin*cII, *Not*I, *Pac*I, *Pvu*II, and *Ppu*MI) (reviewed in Pingoud et al., 2016). The cleavage-deficient and binding-proficient variants (4–8 bp) can be potentially coupled to the 76-aa nicking domain to generate NEases with 9–14 bp nicking specificities. Restriction enzyme catalytic residues can also be predicted among the PD-D/ExK and HNH enzyme superfamilies and catalytic mutants can be constructed with relative ease by site-directed mutagenesis or cloning of mutant gene blocks (Roberts et al., 2003; Pingoud et al., 2016).

Natural NEases have been found as one subunit of restriction endonucleases (e.g., Nb.*Bsr*DI and Nt.BstNBI) (Higgins et al., 2001; Xu et al., 2007), in bacteriophages (phages) and pathogenicity island involved in *cos*/*pac* site cleavage/nicking and DNA packaging (e.g., gp74 of HK97, gp37 of ΦSLT, and Φ12 HNH endonucleases) (Kala et al., 2014), in mobile genetic elements involved in DNA nicking and F element transfer (Luo et al., 1995), in Chlorella viruses that catalyzed the frequent nicking of host genomic DNA (e.g., Nt.CviPII) (Chan et al., 2004, 2006), and in introns encoded homing NEases that catalyze intron mobility [e.g., I-BasI (Landthaler and Shub, 2003), I-TslI (Bonocora and Shub, 2009), I-PfoP3I (Kong et al., 2012), reviewed in (Chan et al., 2011; Xu, 2015)]. NEases can also be engineered from REases and homing endonucleases (HEases) by alteration of catalytic sites (e.g., Nt.*Bbv*CI, Nt.*Bsr*DI) (Heiter et al., 2005; Xu et al., 2007; McConnell Smith et al., 2009) or by nicking domain swapping (Nt.*Alw*I) (Xu et al., 2001).

It is not surprising that the fusion NEase nicks a few bases (N4–6) downstream of the *Bam*HI site. In *Fok*I footprint analysis using methylation protection experiment with dimethyl sulfate (DMS) modification and hydroxyl radical Fe(II)-EDTA foot-printing study, *Fok*I protected ∼3 bp 5′ upstream of its recognition sequence GGATG and some additional bases at the 3′ end (the cleavage domain reaches out and cleaves DNA downstream) (Yonezawa and Sugiura, 1994). It is very likely that most REases occupy and protect a few more bp in addition to their recognition sequences. We found D94N/E113K could partially block *Sma*I digestion of pUC19 plasmid with the overlapping sites in GGATCCCGGG (*Sma*I site = CCCGGG), suggesting *Bam*HI occupies more than 6 bp.

The infrequent NEase reported in this work might be useful for end labeling and DNA optical mapping when its non-specific nicking activity could be minimized. To enhance specific nicking of the composite site, it may be necessary to incubate DNA substrate with the fusion enzyme for 10–20 min to form enzyme-DNA complex and then add divalent cations to activate nicking for a short period of time (5–10 min). Another possibility is to utilize mutants of the 76-aa nicking domain with further weakened DNA binding affinity. Future study and optimization of the infrequent NEase is required prior to any biotechnology applications.

The cleavage-deficient *Bam*HI double mutant may be used in *in vivo* study of DNA binding proteins overlapping a *Bam*HI site or it can serve as a repressor (Dorner and Schildkraut, 1994). As reported previously that *Bam*HI E113K mutant could also block exonuclease progression on linear DNA (Xu and Schildkraut, 1993). Similarly, we expect D94N/E113K binding to *Bam*HI site can also physically block exonuclease degradation of linear DNA. By using similar protein fusion strategy, it is now possible to construct catalytic-deficient PvuRts1I mutant (Type IV REase) and gHNH nicking domain fusion which binds to 5-hydroxylmethylcytocine (5hmC)-modified DNA and introduces nicks near 5hmC modified sites for labeling and detection on mammalian DNA (Janosi et al., 1994).

## Data Availability Statement

The original contributions presented in the study are included in the article/Supplementary Material, further inquiries can be directed to the corresponding author.

## Author Contributions

S-YX designed and performed the experiments, interpreted the data, and wrote the manuscript.

## Conflict of Interest

S-YX is an employee of New England Biolabs, Inc. a company developing restriction enzyme and nicking enzyme reagents for the research and diagnostic community.

## Publisher’s Note

All claims expressed in this article are solely those of the authors and do not necessarily represent those of their affiliated organizations, or those of the publisher, the editors and the reviewers. Any product that may be evaluated in this article, or claim that may be made by its manufacturer, is not guaranteed or endorsed by the publisher.
