# Supplementary material for: Engineering Infrequent DNA Nicking Endonuclease by Fusion of a BamHI Cleavage-Deficient Mutant and a DNA Nicking Domain
Source: Front Microbiol. 2022 Feb 1;12:787073. doi: 10.3389/fmicb.2021.787073 (PMC8845596; doi:10.3389/fmicb.2021.787073)
Supplement: Supplementary file 1 [file Data_Sheet_1.docx]

**Supplementary Material**

**Supplementary Figures S1 and S6.**

**Engineering Infrequent DNA Nicking Endonuclease by Fusion of a BamHI Cleavage-deficient Mutant and a DNA Nicking Domain**

Shuang-yong Xu

New England Biolabs, Inc., 240 County Road, Ipswich, MA 01938

Email: [xus@neb.com](mailto:xus@neb.com)

Telephone: 1-978-380-7287

**Supplementary Figure S1**. **DNA nicking by the fusion nickase in standard restriction buffers (buffer 2, 3 and CutSmart) and in a high salt buffer with nickel cation (100 mM NaCl, 10 mM Tris-HCl, pH 7.5, 1 mM NiCl_2_). A.** pUC19 DNA (1 μg) was used as a substrate in the nicking digestions in buffer 2, 3, and CutSmart buffer (buffer 4 + BSA) at 37^o^C for 1 h. Nb.BsrDI and EcoRI digestions were used as controls for nicked and linear DNA. Low nicking activity was detected in all three buffers (the gel image was overexposed to show the weak nicked products). **B.** Nicking activity in Ni^2+^ buffer for the fusion nickase (0.5, 1, and 2 μg enzyme vs 1 μg DNA). Nb.BsrDI (10 U at 65^o^C for 1 h) nicked DNA served as acontrol. 2-log (1 kb plus), DNA size ladder (0.1 to 10 kb, NEB). NC, L, SC, refer to nicked circular, linear, and supercoiled plasmid DNA, respectively.

**Supplementary Figure S2. Specific activity estimate for gHNH endonuclease (WT), the truncated nicking domain (76 aa) of gHNH endonuclease, and the fusion nickase (BamHI*-gHNH).A.** pUC19 DNA (1 μg) was digested by gHNH endonuclease (0.25 μg) in NEB buffer 3 (lane 1); by the 76-aa nicking domain (0.5 and 1 μg enzyme) in buffer 3 (lanes 2 and 3); by BamHI*-gHNH fusion in buffer 3 supplemented with 1 mM MnCl_2_ (0.5, 1, 2 μg, lanes 4-6 and lanes 7-9); lane 10, uncut DNA. The fusion nickase lot 2 and lot 3 refer to two separate enzyme preparations from chitin column chromatography. The specific activity was estimated at approximately 1-2 x 10^3^ U/mg for BamHI*-gHNH fusion. The 76-aa truncated nicking domain only achieved partial nicking due to its attenuated activity (estimated at less than 500 U/mg). **B.** Enzyme titration for gHNH endonuclease (WT) in nicking 1 μg of pUC19 DNA in NEB buffer 3 at 37^o^C for 1 h. Some smaller fragments and linear form were also detected due to nicking of two closely located nicking sites on both strands at high enzyme concentration. The specific nicking activity was estimated at 4-8 x 10^3^ U/mg (i.e. complete nicking at 0.125 μg enzyme vs 1 μg DNA, lane 5).Nb.BsrDI (10 U at 65^o^C for 1 h) nicked pUC19 DNA served as a control. 2-log (1 kb plus, 0.1-10 kb), DNA size ladder. NC, SC, and L refer to nicked circular, supercoiled, and linear DNA respectively.

**Suppl. Fig. S3. Example of DNA run-off sequencing: mapping of BspQI (GCTCTTC N1/N4) cut site. A.** BspQI is a Type IIS REase that cuts downstream of its recognition sequence 5’GCTCTTC3’. **B.**The bottom strand is cleaved at the N4↑ position and therefore Taq DNA polymerase added an extra “A” peak after the broken template and the sequencing peaks suddenly drop off after the high “A” peak (bottom panel). If the pBR322 were partially digested by BspQI, a doublet (A/T) would be detected in the run-off sequencing and the peaks after the doublet would be flatten out. The top-strand cut site at N1↓ position was marked by a high “T” peak (top panel). If the BspQI digestion were partial, a doublet (T/C) would be detected at the cut site. **C.**Sequencing of two strands of uncut DNA. **D.**Raw sequencing data of bottom-strand read from cleaved top strand template. The appearance of extra high “A” peak (sequencing of cleaved bottom strand) or “T” peak (sequencing of cleaved top strand) or strong doublets in the run-off sequencing reactions are indications of cleavage of the DNA templates.

**Suppl. Fig. S4. DNA run-off sequencing of BamHI partially digested pUC19 near BamHI site.** Oneμg of pUC19 DNA was digested by 5 and 1 U of BamHI endonuclease respectively for 20 min at 37^o^C to achieve partial digestions (20 U in 1 h would have accomplished complete digestion). The digested DNA was treated at 95^o^C for 6 min to inactivate BamHI and the DNA was subjected to Sanger sequencing. The BamHIcut top-strand was sequenced to get the bottom strand sequence. The strong A/C doublet indicated the cut position within the BamHI site. The same sequences are shown in reverse complement (panels 4-6). The appearance of an extra “T” peak in the T/G doublets indicated cleavage at the 5’ G↓GATC 3’ site (panels 5 and 6). The height of T peak indicated the extent of the partial digestion (the lower “T” peak in the T/G doublet implied more partial digestion, the high “T” peak in the T/G doublet implied nearly complete digestion). Panels 1 and 4 are sequencing reads from uncut pUC19 control. See the top and bottom strand definition of pUC19 sequence in Materials and Method.

**Suppl. Fig. S5. Digestions of a pUC19 derivative with BamHI site deletion (pUC19-ΔBamHI).** Plasmid pUC19-ΔBamHI (1 μg prepared from NEB 10β cells) was digested by BamHI*-gHNH fusion enzyme (lot 2, 2, 1, 0.5, 0.25 μg) for 1 h at 37^o^C in NEB buffer 3 supplemented with 1 mM MnCl_2_ (lanes 1-4), by TE buffer (lane 5), by BamHI (10 U, lane 6),EcoRI (10 U, lane 7), and Nb.BsrDI (10U, 65^o^C, lane 8) digestions, respectively. BamHI failed to cleave this plasmid due to the loss of BamHI site. EcoRI digested this plasmid to linear DNA; Nb.BsrDI generated nicked circular DNA. Lane 9, 2 log DNA ladder (1 kb plus, 0.1 to 10 kb). SC, L, NC: supercoiled, linear, and nicked circular DNA, respectively. The size of linear pUC19 DNA is ~2.7 kb. Lanes 10-11, pUC19 (BamHI^+^) digested by the fusion nickase(lot 2, 2 and 1 μg) for 1 h at 37^o^C in NEB buffer 3 supplemented with 1 mM MnCl_2_. Lane 12, uncut pUC19.

**Supplementary Figure S6**. **DNA run-off sequencing of nicked products from nicking digestions carried out in high salt buffers (100 to 200 mM NaCl/KCl)**. Plasmid pUC19 was digested by the fusion enzyme for 30 min in the high salt buffer shown on the left of the figure (NEB buffers 2 or 3 supplemented with 50 to 100 mM KCl). The nicked DNA was purified by spin columns (Qiagen) and subjected to DNA run-off sequencing (forward top-strand read). The up arrows indicate the nicking site AC↑CGA where the bottom strand was nicked as evidenced by the appearance of an extra “A” peak (i.e.A/C doublet). Del1-4 indicate regions where the intervening sequences have been deleted to show the downstream sites (ACCGA, ACCGC, ACCGT, and ACCGA) that are not immediately adjacent to the BamHI site. The undigested pUC19 serves as the negative control for the forward sequencing reaction. BigDye™ terminator v3.1 cycle sequencing kit (Thermo-Fisher/ABI) was used for DNA sequencing reactions. To prevent nicking at the unintended sites, it is recommended to carry out the nicking reaction in a high salt buffer (100 to 200 mM NaCl/KCl) for a short time(30 min) to minimize star activity.
